# Sensitivity of two SARS-CoV-2 variants with spike protein mutations to neutralising antibodies

**DOI:** 10.1007/s11262-021-01871-8

**Published:** 2021-10-04

**Authors:** Katharina Müller, Philipp Girl, Andreas Giebl, Stefanie Gruetzner, Markus Antwerpen, Elham Khatamzas, Roman Wölfel, Heiner von Buttlar

**Affiliations:** 1 Bundeswehr Institute of Microbiology, Munich, Germany; 2 German Centre for Infection Research (DZIF), Partner site Munich, Munich, Germany; 3 Medical Faculty, Institute for Transfusion Medicine and Haemostasis, University of Augsburg, Augsburg, Germany; 4 Department of Medicine III, LMU Hospital Munich, Munich, Germany; 5 Present Address: Heidelberg University Hospital, Heidelberg, Germany

**Keywords:** COVID-19, SARS-CoV-2, Variants of concern, B.1.351, Mutation E484K, Neutralising antibodies, Immune escape

## Abstract

SARS-CoV-2 infections elicit a humoral immune response capable of neutralising the virus. However, multiple variants have emerged with mutations in the spike protein amongst others, the key target of neutralising antibodies. We evaluated the neutralising efficacy of 89 serum samples from patients, infected with SARS-CoV-2 in the beginning of 2020, against two virus variants isolated from acutely infected patients and harbouring spike protein mutations. One isolate was assigned to lineage B.1.351 (MUC-IMB-B.1.351) whilst the other (MUC-484) was isolated from an immunocompromised patient, sharing some but not all mutations with B.1.351 and representing a transitional variant. Both variants showed a significant reduction in neutralisation sensitivity compared to wild-type SARS-CoV-2 with MUC-IMB-B.1.351 being almost completely resistant to neutralisation. The observed reduction in neutralising activity of wild-type-specific antibodies against both variants suggests that individual mutations in the spike protein are sufficient to confer a potent escape from the humoral immune response. In addition, the effect of escape mutations seems to accumulate, so that more heavily mutated variants show a greater loss of sensitivity to neutralisation up to complete insensitivity as observed for MUC-IMB-B.1.351. From a clinical point of view, this might affect the efficacy of (monoclonal) antibody treatment of patients with prolonged infections as well as patients infected with variants other than the donor. At the same, this could also negatively influence the efficacy of current vaccines (as they are based on wild-type spike protein) emphasising the need to thoroughly surveil the emergence and distribution of variants and adapt vaccines and therapeutics accordingly.

## Introduction

SARS-CoV-2 has steadily changed its genome through mutations during its global spread amongst humans. Such mutations can in principle affect the entire viral genome. Of particular interest, though, are those that occur in the spike (S) protein are of particular interest, since this protein is responsible for binding to the human ACE2 receptor and is thus indispensable for virus cell entry. It is therefore not surprising that the S protein and in particular its receptor binding domain has been identified as the key target for eliciting potent neutralising antibodies (NAbs), likely protecting against reinfection [1, 2]. Moreover, the therapeutic administration of monoclonal antibodies (mAbs) and convalescent plasma from recovered patients is successfully applied worldwide.

However, several novel variants that have recently emerged harbour a number of mutations, some of which are in the gene encoding the S protein [3]. This has sparked questions about their humoral immune evasion potential and the protective capability of wild-type (WT)-specific NAbs against such virus variants. In this context, we analysed serum samples from convalescent plasma donors infected with SARS-CoV-2 at the onset of the pandemic (April–May 2020) and found a significant reduction in neutralising activity (47.7%) when tested against Variant of Concern (VOC) B.1.1.7 (Alpha), confirming humoral immune evasion, at least to some extent [4]. To investigate this finding further, we reevaluated these samples and tested their neutralising effect on another VOC, B.1.351 (Beta), isolated from an acutely infected patient (MUC-IMB-B.1.351). In addition, we also tested the neutralising capability of WT-specific NAbs against a SARS-CoV-2 strain (MUC-484), which originated in an immunocompromised patient during the course of a prolonged infection with multiple therapeutic plasma donations and was first described by Khatamzas et al. [5]. The previously described and early circulating strain MUC-IMB-1 (clade B1) served as WT reference virus [6].

Lineage B.1.351 (Beta), also known as 20H/501Y.V2, is one of multiple SARS-CoV-2 variants circulating globally and is classified as a VOC by the WHO [7]. It was first identified in South Africa and whilst it shares some mutations with B.1.1.7, it emerged independently [8]. It has a well described asparagine to tyrosine substitution (N501Y), which is also present in VOCs belonging to the B.1.1.7 and P.1 lineages [9]. In addition, it also hosts two other mutations E484K and K417N, both located in the ACE2 interaction surface of the S protein [10]. Interestingly, E484K was originally absent in B.1.1.7 including the strain we tested in our previous study. It was not until later that some B.1.1.7 isolates also acquired this particular mutation (commonly referred to as “B.1.1.7 enhanced”) which was shown to reduce the sensitivity to WT Nabs of these enhanced isolates, allowing for an even greater humoral immune evasion [9]. Current studies suggest that this variant is more transmissible, likely due to carrying the early described D614G mutation associated with increased infectivity and has a significantly reduced susceptibility to various monoclonal antibody treatments as well as NAbs [11–14].

MUC-484 has several mutations, four of which are located within the S protein, including the D614G mutation. Most notably, the strain also acquired the E484K mutation present in B.1.351. However, it contains neither the above-mentioned N501Y nor the K417N mutation, making it an interesting variant to investigate the influence of E484K in absence of N501Y and K417N compared with the more recent line B.1.351, which contains all three mutations.

## Materials and methods

### Origin of serum samples

As previously described [4], all samples were from patients which were hospitalised for their SARS-CoV-2 infection, but did not require mechanical ventilation. All samples were collected, according to the official guidelines of the German Federal Institute for Vaccines and Biomedicine [15] for the selection of plasma donors, 4 weeks after complete recovery. All had a serological profile consistent with recent SARS-CoV-2 infection (IgA/IgG antibodies, data not shown). Initially, 89 samples were tested against MUC-IMB-1 and MUC-B.1.351. However, nine samples were completely used up in the process, leaving only 80 samples to be tested against MUC-484.

### Isolation of SARS-CoV-2 variants

Replication competent SARS-CoV-2 was isolated from a nasopharyngeal swab of a patient diagnosed with COVID-19 by quantitative PCR with reverse transcription (RT-qPCR). Whole genome sequencing of this isolate (from now on referred to as MUC-IMB-B.1.351) was performed in accordance with the German Regulation for molecular genetic surveillance of the coronavirus SARS-CoV-2. The second isolate (from now on referred to as MUC-484) derived from an immunocompromised patient and was previously described [5]. NGS sequencing and phylogenetic analyses of both SARS-CoV-2 isolates were performed as previously described [16]. Lollipop plots were subsequently generated using R in combination with the trackViewer libaray [17].

Both viruses were grown on Vero E6 cells. Viral stocks were prepared, titrated and stored at − 80 ℃ until further use.

### Micro-neutralisation test

SARS-CoV-2 NAb titres were determined as previously described [18]. In brief, serum samples were serially diluted in duplicate in 96-well tissue culture plates starting at 1:5 to a maximum if 1:640 along with positive and negative control samples. Virus stocks (50 TCID/50 µl) of B.1.351, MUC-484 and MUC-IMB-1 were prepared on Vero E6 cells; aliquots were stored at − 80 ℃ until further use. Each Virus was pre-incubated (1 h, 37 ℃) with diluted samples before Vero E6 cells (1 × 10^4^ cells/50 µl) were added to each well. After 72 h (37 ℃), supernatants were discarded and wells were fixed (13% formalin/PBS) and stained with 0.1% crystal violet. The NAb titre corresponded to the reciprocal of the highest sample dilution showing complete inhibition of CPE. A virus re-titration was performed in triplicates on every plate and exact titres were determined by retrograde calculation.

## Results

### Mutational changes in MUC-IMB-B.1.351

**Fig. 1:**
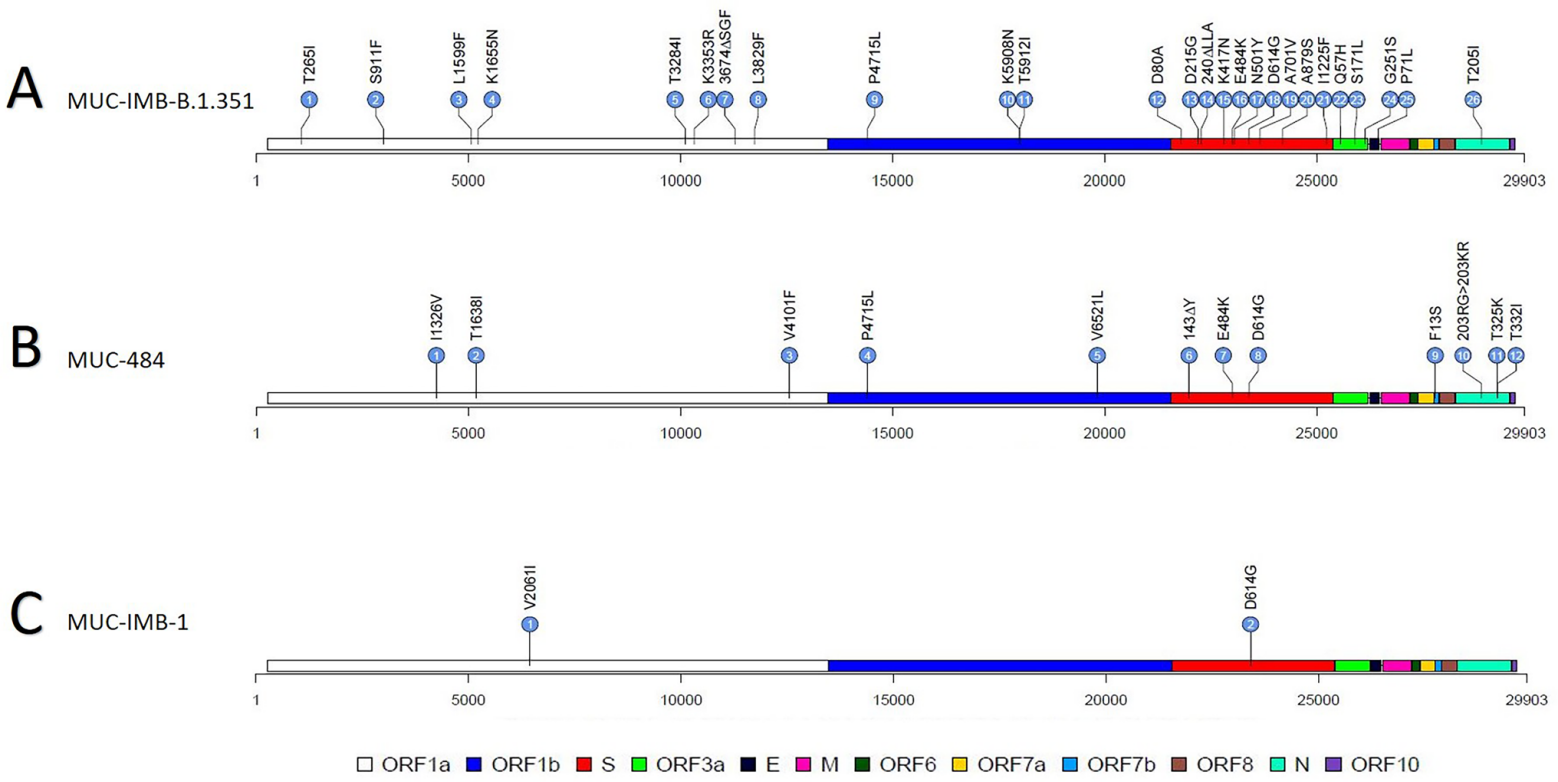
Lollipop plots of all three SARS-CoV-2 strains examined in this study showing all nonsynonymous mutations in their respective genomes. **A** MUC-IMB-B.1.351 holds 26 mutations distributed over the genome. **B** MUC-484 shares only a few mutations with lineage B.1.351. **C** MUC-IMB-1 was used as WT SARS-CoV-2 reference strain

We were able to generate a high-quality genome from a patient sample containing replication competent SARS-CoV-2. The isolated genome holds 29,903 nucleotides in length, which equals 100% of the SARS-CoV-2 genome. The theoretically obtained sequencing depth was 1536-fold. Sequencing analysis revealed 26 nonsynonymous mutations relative to the Wuhan sequence, assigning the isolate to the lineage B.1.351. An overview of all mutations is shown in Fig. 1A. Most notably, ten of the 26 mutations are located within the S protein. Prominent S-specific mutations include the amino acid changes K417N, E484K, N501Y and D614G. Moreover, amino acid changes D80A, D215G as well as the in-frame deletion 240∆LLA are present in the N-terminal domain (NTD) of the S protein.

As shown in Fig. 1B, MUC-484 contains only three nonsynonymous mutations in the S protein, including D614G, E484K and an in-frame deletion (143∆Y). In contrast to both variants, reference strain MUC-IMB-1 only has two nonsynonymous mutations present in its entire genome, one of which is located in the S protein (D614G) and also present in the other strains used in this study (Fig. 1C).

### Sensitivity of MUC-IMB-B.1.351 to sera from convalescent plasma donors

**Fig. 2:**
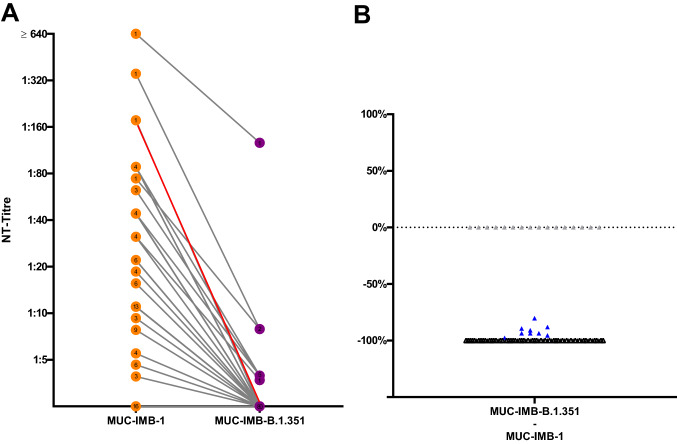
Comparison of MUC-IMB-1 and MUC-IMB-B.1.351 demonstrates a significant decrease in sensitivity towards NAbs (**A**) Side by-side comparison of NAbs titres against WT (orange) and MUC-IMB-B.1.351 (purple). The greatest decrease was observed in a sample, which dropped from an initial titre of 177 to no neutralising effect against B.1.351 (red). (**B**) All but nine samples (blue) show a complete loss of neutralising activity (black). All samples that tested negative for NAbs against WT remained negative when tested against MUC-IMB-B.1.351 (grey). (∑ samples: 89, the number of overlapping samples is shown in the respective circle)

Of the 89 samples tested, 73 were able to neutralise WT SARS-CoV-2 whilst for the remaining 16 samples no neutralising activity could be observed. Direct comparison of NAbs titres against MUC-IMB-1 and MUC-IMB-B.1.351 revealed that the vast majority of these positive serum samples (64/73) was unable to neutralise MUC-IMB-B.1.351. For the nine samples that partly retained their neutralising ability, a notable reduction was observed with a median decrease of 91.5% in neutralising activity. Altogether, the mean loss of neutralising activity against MUC-IMB-B.1.351 was 99% and the observed decrease in titres was statistically significant (*p* < 0.0001 by Wilcoxon). At the same time, all samples (16/16) that initially tested negative for NAbs against WT virus were also unable to neutralise MUC-IMB-B.1.351 (Fig. 2).

**Fig. 3:**
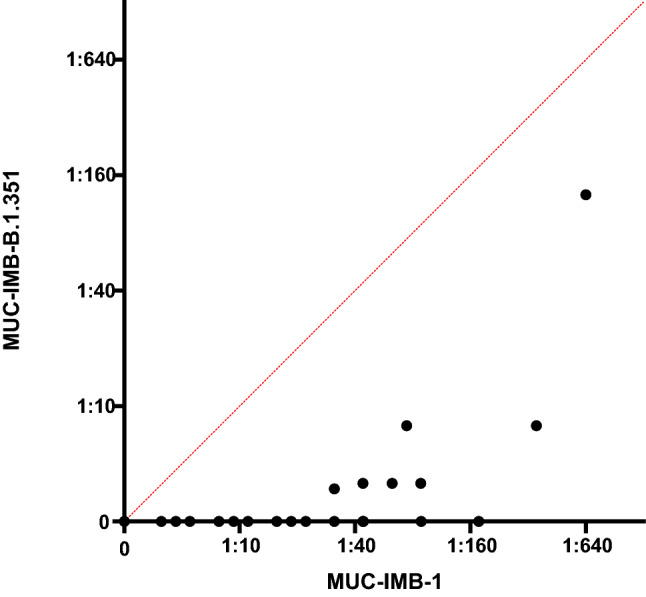
Correlation plot reveals limited correlation between NAbs titres against MUC-IMB-1 and MUC-IMB-B.1.351 No prediction can be made about the neutralising efficacy of a serum against MUC-B.1.351 based on the NAbs titre of the same sample against WT SARS-CoV-2 and vice versa (*r* = 0.53, 95% CI: 0.37–0.67 Pearson regression analysis)

Pearson regression analysis revealed only limited correlation (*r* = 0.53; 95% CI: 0.37–0.67) between the NAb titre against WT SARS-CoV-2 and the titre against MUC-IMB.1.351 of the same sample. (Fig. 3).

### Sensitivity of MUC-484 to sera from convalescent plasma donors

**Fig. 4:**
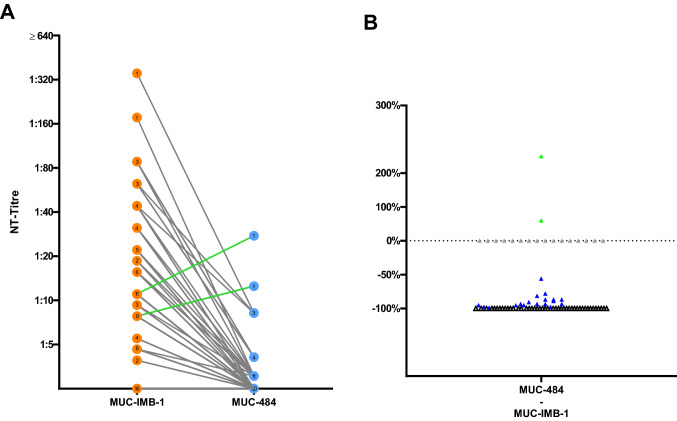
Comparison of MUC-IMB-1 and MUC-484 demonstrates an overall decrease in sensitivity towards NAbs (**A**) Side-by-side comparison of WT (orange) and MUC-484 (blue) reveals an overall decrease in NAbs titre levels with the exception of only two samples depicted in green, showing a lowgrade increase in titre level from 11 to 27.6 and 7.8 to 12.5, respectively. (**B**) Whilst one third of samples was still able to neutralise MUC-484 (blue), a notable decrease in titre levels can be observed for all but two of those samples with a median decrease of 97.1%. Two samples show an increase in neutralising activity (green). The remaining two-thirds of the samples show a complete loss of neutralising efficacy against MUC-IMB-1 (black) whilst all 16 samples that tested negative for NAbs against MUC-IMB-1 remained negative when tested against MUC-484 (grey). (∑ samples: 80; the number of overlapping samples is shown in the respective circle)

Direct comparison of NAbs titres against WT and MUC-484 showed that the majority of the tested serum samples (∑ = 80) was unable to retain their neutralising efficacy. Of the 64 samples that were able to neutralise MUC-IMB-1 only one-third (20/64) was also able to neutralise MUC-484. At the same time, a notable reduction in NAbs titres was observed for 18 of these samples with a median decrease of 89.6%. Two samples showed a slight increase in neutralisation when tested against MUC-484. A total of 44 samples showed no neutralisation when tested against MUC-484 although they were able to neutralise WT SARS-CoV-2 in parallel control tests. Altogether, the median loss of neutralising efficacy was 90.6%. All 16 samples that initially tested negative for NAbs against WT virus were also unable to neutralise MUC-484 (Fig. 4A & B). Overall, the observed drop in NAbs titres against MUC-484 compared to WT virus was statistically significant (*p* < 0.0001 by Wilcoxon).

**Fig. 5:**
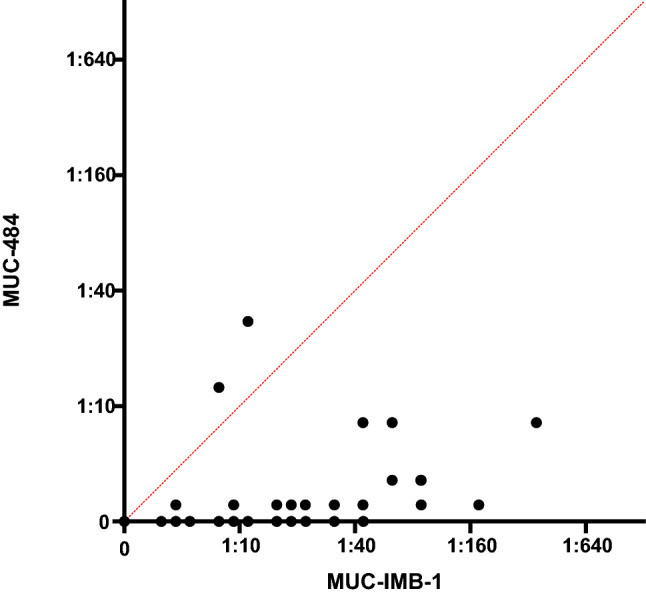
Correlation plot reveals very limited correlation between NAbs titres against MUC-IMB-1 and MUC-484 Based on the very limited correlation, no predictions can be made about the neutralising effect of a serum against MUC-484 based on the titre of the same sample against WT SARS-CoV-2 (*r* = 0.37, 95% CI: 0.17–0.55 Pearson regression analysis)

Pearson regression analysis revealed very limited correlation (*r* = 0.37; 95% CI: 0.17–0.55) between the NAb titre against WT SARS-CoV-2 and the titre against MUC-IM-B.1.351 of the same sample (Fig. 5). As the changes in titre were highly variable, no prediction could be made about the neutralising efficacy of a serum against MUC-484 based on the titre against WT SARS-CoV-2.

## Discussion

We investigated the sensitivity of an early circulating SARS-CoV-2 and two mutation variants to sera from long-term convalescent plasma donors infected between April and May 2020. Both variants harbour diverse mutations in the gene encoding the S protein, raising concerns about their humoral immune evasion potential. We used clinical viral isolates instead of pseudoviruses to include the effects of potentially relevant mutations outside the S protein. We isolated MUC-IMB-B.1.351 from an acutely infected individual. Whole genome sequencing assigned the isolate to lineage B.1.351 and revealed ten nonsynonymous mutations in the S protein relative to the original Wuhan sequence. MUC-484 was isolated from an immunocompromised patient with a prolonged SARS-CoV-2 infection and was first described by Khatamzas et al. [5]. As shown, it holds three nonsynonymous mutations in the S protein. Early isolated MUC-IMB-1 was used as WT reference strain with only two mutations in total, one of which is located in the S protein.

Substitution D614G is the only S mutation that is present in all three strains examined in this study including the WT reference strain. It could therefore not be assessed with regard to its influence on neutralisation sensitivity. However, numerous studies suggest that the D614G mutation increases overall fitness and infectivity but does not seem to promote immune evasion [19, 20]. Substitutions K417N, E484K, N501Y are commonly associated with VOCs and are of special interest as all of them have been shown to efficiently mediate antibody escape [13, 21]. MUC-484 only acquired the E484K mutation and was highly resistant to convalescent sera in our study with a median loss of neutralising efficacy of 90.6%. For comparison, we found a median decline of 99% for MUC-IMB-B.1.351 carrying all three substitutions. These findings underline that the E484K mutation alone is vigorously reducing neutralisation sensitivity due to its enhancement of hACE2 binding whilst reducing the binding affinities of neutralising monoclonal antibodies [22]. At the same time, this also indicates that even a small number or even a single mutation in the S protein may be sufficient to significantly reduce antibody-mediated immune protection against SARS-CoV-2. This is also well in line with our recent report on the significant reduction of neutralising activity of the same sera against B.1.1.7. The observed decrease of 47.7% was less than the reduction we found for the variants tested in this study [4], which is likely because B.1.1.7 misses the vigorous E484K mutation and the observed antibody escape is mediated by N501Y only. Our observation that MUC-IMB-B.1.351 is even more resistant to neutralisation could be explained by the accumulation of additional substitutions (including K417N and N501Y). It could also be an indication that S mutations act synergistically and their effect on each other is enhanced. In addition, the impact of NTD-specific antibodies on virus neutralisation has been described [23]. MUC-IMB-B.1.351 carries mutations located within the NTD (i.e. two substitutions and one in-frame deletion) that were previously shown to influence NTD-specific NAbs [24]. Based on our findings, these mutations likely contribute to the neutralisation resistance of MUC-IMB-B.1.351 but appear to play a minor role.

In contrast, we were surprised by the finding that two sera showed increased neutralising activity against MUC-484 (but not MUC-IMB-B.1.351). Interestingly, we observed the same phenomenon with one serum when tested against B.1.1.7 [4]. This could indicate that in rare cases polyvalent antibody formation could also improve neutralisation against variants. However, this could be contradicted by the fact that this effect was observed in different samples in each case. This is consistent with structural predictions showing that K417N and E484K inhibit the binding of different monoclonal antibodies and can therefore act synergistically in a polyclonal serum [22].

The fact that the E484K emerged independently in an immunocompromised patient with prolonged infection under plasma donation treatment [5] and is highly resistant to neutralisation is particularly striking. It highlights the problem that it is not only necessary to monitor the emergence of variants at the population level, but also for individual patients in some circumstances. Especially in severe and longstanding COVID-19 cases, treatment with convalescent plasma seems to promote the emergence of escape variants and decrease the efficacy of plasma therapy over time [25, 26].

The fact that no prediction could be made about the neutralising efficacy of a serum against either variant based on the NAbs titre of the same sample against WT SARS-CoV-2 must be considered, especially for the therapeutic use of convalescent plasma. Ideally, the neutralising efficacy of convalescent plasma should be determined against the major virus clades circulating at that time and administered to patients infected with a well-neutralised clade whenever possible. This includes that NAbs detection assays such as virus neutralization tests (VNT) or surrogate tests should be continuously adapted to cover current virus clades.

A potential limitation of our study is its relatively low number of tested samples as well the overall low titres of these samples, which might lead to an overestimation of the variants immune escape potential In addition, we could not include in our investigations the potentially pre-existing cellular immune response, which might be more cross-reactive than NAbs. Thus, future studies focussing on the cellular immune response are needed.

Overall, our study highlights the humoral immune evasion potential not only of globally circulating VOCs such as B.1.351 but also of variants emerging in patients under plasma therapy. Mutations of the S protein are of particular interest and we demonstrate that different mutations and combinations thereof can be associated with a reduction up to complete loss of neutralising antibody cross-reactivity against novel emerging viral strains.

## Data Availability

The data presented in this study are available on request from the corresponding author. The data are not publicly available due to privacy restrictions.
